# Salivary Distinctiveness and Modifications in Males with Diabetes and Behçet's Disease

**DOI:** 10.1155/2017/9596202

**Published:** 2017-02-21

**Authors:** Loai Aljerf, Iyad Alhaffar

**Affiliations:** ^1^Department of Life Sciences, Faculty of Dentistry, University of Damascus, Damascus, Syria; ^2^Department of Oral Medicine, Faculty of Dentistry, University of Damascus, Damascus, Syria

## Abstract

Oral diseases associated with systematic diseases as metabolic and vasculitic have been included in this paper. This will enhance our understanding of the salivary function in promoting healthy oral condition. The study investigates the effects of type I and type II diabetes mellitus in well-controlled diabetic patients, in addition to* Behçet disease (BD)* on saliva flow rate (SFR), pH, the decay, missing, and filled tooth (DMFT) index, glucose, and major earth-alkaline ions (Ca^2+^ and Mg^2+^) compared to healthy males and age-matched controls. Saliva samples were collected from 1403 male human subjects, distributed on 7 levels including 3 control groups, and analyzed. The symptoms and clinical observations were enrolled. A preprandial salivary glucose has illustrated statistically strong significant and positive correlations with HbA_1c_ and blood glucose levels. TIDM saliva showed lower pH, SFR, and Ca^2+^ but higher Mg^2+^, caries risk, and poor metabolic control. These led to dysfunction of secretory capacity of salivary glands. TIIDM proved higher SFR, DMFT, and glucose than TIDM patients. DM oral calcium has decreased by age while magnesium sharply slopes at seniority. BD oral fluid is associated with lower glucose and minerals but noticeably with both higher pH and DMFT.

## 1. Introduction

Most commonly used laboratory diagnostic procedures involve analyses of the cellular and chemical constituents of blood. Despite that, saliva offers some distinctive advantages over the other biological fluids. Collection and evaluation of the secretions from the individual salivary glands are primarily useful for detection of gland-specific pathology, that is, infection and obstruction.

The understanding of salivary function in promoting healthy oral condition has become a topic of major importance for the nowadays-oral clinicians. In order to comprehend the role of each salivary component in oral cavity homeostasis, it is crucial to perceive how the changes of these components or their absences may be linked with pathological conditions [1]. However, knowledge integration between saliva and oral pathology is far from being complete. Therefore, it is of critical importance to establish which salivation patterns and concentration ranges of each salivary component are to be considered as normal in order for the clinician to diagnose altered salivary phenotypes possibly linked to pathological systemic or oral conditions [2]. Several systemic diseases have been reported to produce marked and identifiable salivary changes as metabolic, that is, diabetes [3], and vasculitic, that is,* Behçet* (BD) [4].

*Diabetic* is a wide spread metabolic disease causing well-documented deleterious effects on general health [2]. It is also probably the most frequent metabolic disease with salivary implications. Salivary hypofunction and increased susceptibility to oral infections as caries or periodontitis have long been recognized features of diabetes mellitus (DM) [5], particularly when there has been dehydration and inadequate glucose (C_6_H_12_O_6_) blood control [6]. However, there is little knowledge concerning the true effects of diabetes on salivary parameters of well-controlled patients and the way that the two types of this disease affect their patients. DM is a chronic disease resulting from a relative or absolute deficiency of insulin (C_257_H_383_N_65_O_77_S_6_), which affects the metabolism of carbohydrate (C_m_(H_2_O)_n_), protein, and fat. The most obvious abnormality is a high level of blood glucose especially following a meal. According to the data issued by the World Health Organization DIAMOND Project Group, the number of children with DM is continually increasing [7]. The current concept in diabetic care with blood glucose monitoring and frequent injections of short-acting insulin allows a less restricted diet [8]. This may affect oral health rapidly and as a result requires attention. However, there are many internal and external factors that contribute to DM and in turn affect the general health and more so oral health [9].

*BD* was first described by the Turkish dermatologist* Hulusi Behçet* in 1937. This disease was known as “triple-symptom complex” carrying signs similar to those described by Adamantiades [10]. Cases of BD have been found along the path of the Silk Road, a 4000-mile long ancient trading route that spanned from Mediterranean basin to China [4]. Prevalence varies in countries along the path, ranging from 2 to 370 per 100,000 with highest occurrence ratios noted in Turkey and Japan [11]. Prevalence in non-Silk Road countries such as United States is substantially lower at 0.33 per 100,000 [12]. This disease is rarer in children who encompass only 2-3% of all cases of BD [12]. Risk increases with human lymphocyte antigens (HLA) B5 or B51 [13]. However, HLA B51 carries the risk of BD at 6.3 [13].

The grounds of BD remain elusive, although many theories have been suggested [14]. Behçet [10] proposed that herpes virus is the cause of the disease. There is evidence showing a greater presence of herpes simplex virus DNA in BD patients' saliva. Lee et al. [15] showed that nearly 60% of these patients have no herpes simplex virus isolated. The most common symptoms of BD in adult and pediatric cases are oral aphthous ulceration (95%–100%) and uveitis (30%–75%) [12]. However, when compared to adults, children are less likely to have genital ulcers and vascular lesions and even are more likely to have arthralgia and central nervous system (CNS) symptoms [16].

Recently, there are some researchers [17–19] who proposed the possibility of using saliva as an alternative to blood in diagnosing and monitoring of diseases. However, the knowledge of the effects of types I and II DM and BD on salivary functions remain equivocal, although there are unsatisfactory studies that have been conducted in this regard. It is currently essential to study whether the salivary physical and biochemical characteristics would be altered in these diseases.

In this study, we have investigated the effects of types I and II DM in well-controlled diabetic patients and BD on the quality and quantity of saliva compared to health age-matched male participants. Thus, we aimed to evaluate saliva flow rate (SFR), pH, the decay, missing, and filled tooth (DMFT) index, glucose, and major earth-alkaline ions (Ca^2+^ and Mg^2+^) in the saliva of candidate patients, since studies related to these issues are scant and their results are inadequate.

## 2. Materials and Methods

### 2.1. Standard Materials

To establish the ions calibration series, standard materials of certified purity of freeze dried urine (SRM 2670, NIST, Gaithersburg, MD, USA) and artificial saliva prepared in our lab were all being equipped and used. Eight-point calibration approach and stock solutions were prepared containing 0.2, 0.5, 1.1, 1.5, 2.1, 2.6, 3.1, and 3.9 mM Ca^2+^ and 0.1, 0.2, 0.3, 0.5, 0.6, 0.7, 0.9, 1.0 mM Mg^2+^. All standard solutions were prepared in polystyrene test tubes and stored in 500 *μ*L portions in polypropylene vials at −20°C (−4°F) and discarded after two freeze-thaw cycles.

### 2.2. Apparatus for Earth-Alkaline Measurements

A Microlyte 6 analyzer (Thermo Fisher Scientific Oy, Vantaa, Finland) equipped with calcium selective electrode based on solid-state PVC polymer using phenylene bis(ditolyl) phosphine oxide as ionophore and magnesium selective electrode based on tetraphenylborate salt of Mg-4,7-diphenyl-1,10-phenanthroline (1 : 3) complex in *o*-nitrophenyl octyl ether in PVC matrix were implemented.

### 2.3. Ethics

The study was done in accordance with the Declaration of Helsinki, good clinical practice, and International Organization for Standardization standard 14155. The protocol was approved by the institutional ethics committees at our institution. All patients provided written informed consent. Completeness and quality of data were assured by 100% source document verification. An independent data monitoring committee adjudicated all adverse clinical events. So, the eligible patients signed their approvals to participate in this study and the Institutional Ethics Committee of Faculty of Dentistry/University of Damascus (IEC-FD-DU) had provided participants the ethical clearance and all information regarding risks and benefits of this research. Then, consent was gathered. On the other hand, parents of healthy children and patients of TIDM gave written informed consent. Then, individuals' pro forma was prepared to gather adequate information from case sheets of patients including symptomatology and laboratory investigation.

### 2.4. Patients Selection and Categorization

Patients were males and had been chosen on the basis that all of them were permanent residents in Damascus and living far from plaster dust sources (incl. concrete plants) of any possible adjacent construction sites and they were nonsmokers from nonsmoker families. This procedure was reported as consensus findings including further recommendations of the National Committee of Environmental Health Sciences (NCEHS) expert panel. These important conditions were highlighted in the patient's medical record which also included the glycosylated hemoglobin (HbA_1c_) data.

This research had selected the unstimulated whole saliva for control and infectious samples. The whole saliva specimens were chosen and collected since this type of saliva predominates during most part of the day and is usually considered more important for the maintenance of oral health, reflecting a physiological status of oral cavity and of entire body. Moreover, 3 types of oral diseases, type I diabetes mellitus (TIDM), type II diabetes mellitus (TIIDM), and BD, were involved in this study and their main results of DMFT index, SFR, glucose, and major earth-alkaline ions had been compared with the corresponding outcomes of control samples. This work was utterly executed in the Faculty of Dentistry, University of Damascus/Damascus.

Seven levels of whole saliva samples (*N* = 1403) were collected from male children.* Level 1*: unstimulated saliva samples (age: 2–15 yrs, mean age (*μ*_age_) ± standard deviation (SD): 8.2 ± 4.15 yrs) were selected from 40 healthy and nondiabetic young individuals (*N* = 40). None of this group had systemic diseases or any local infection before 3 months and did not also take any medication for at least 3 months before saliva collection. Dental examinations were conducted by a paedodontist under natural light. Children with congenital oligodontia and delayed eruption (more than 1 yr) were excluded. All erupted teeth were evaluated according to the criteria recommended by World Health Organization (WHO) [20].* Level 2*: unstimulated saliva samples (age: 20–39 yrs, *μ*_age_ ± SD: 28.5 ± 9.04 yrs) from 215 healthy and nondiabetic young individuals (*N* = 215) were selected. The inclusion criteria of group selection were also having no symptoms of any disorder and persons who had not used orthodontic apparatuses or taken any medicine during last month and had similar socioeconomic status.* Level 3*: unstimulated saliva samples (age: 40–60 yrs, *μ*_age_ ± SD: 51.2 ± 8.96 yrs) from 306 healthy and nondiabetic young individuals (*N* = 306) were selected. The inclusion criteria of group selection were also having no symptoms of any disorder and persons who had not used orthodontic apparatuses or taken any medicine during last month and had similar socioeconomic status.* Level 4*: TIDM individuals (*N* = 95) (age: 5–15 yrs, *μ*_age_ ± SD: 11.3 ± 2.05 yrs) were selected from the public health pediatric endocrinology service (PHPES) in the Faculty of Dentistry. Inclusion criteria for patients of this level were students living in the same region, with similar socioeconomic status.* Level 5*: unstimulated saliva samples from 327 type II young diabetic patients (*N* = 327) (age: 20–39 yrs, *μ*_age_ ± SD: 29.2 ± 8.3 yrs) were gathered. The diabetic subjects were consecutive patients attending the Endocrine Unit of the Medical Out-Patients Department (EUMOPD). The enrolled cases had a history of TIIDM for a minimum duration of 2 yrs. Subjects did not take any caries-preventive regimen like fluoride toothpaste, fluoride rinses, or sodium fluoride (NaF)/calcium tablets. However, participants who were on treatment with antidepressants, antihistaminic, and antihypertensive, were edentulous or had any systemic illnesses, or underwent radiotherapy to head and neck regions were excluded from the study.* Level 6*: 364 TIIDM subjects (*N* = 364) (age: 40–60 yrs, *μ*_age_ ± SD: 48.7 ± 6.2 yrs) were consecutive patients attending the EUMOPD. The enrolled cases had a history of TIIDM which had a minimum duration of 2 yrs. Participants who were on treatment with antidepressants, antihistaminic, and antihypertensive were excluded. In addition, participants who were edentulous or had any systemic illnesses and those who were undergoing radiotherapy to head and neck region were also excluded from the study.* Level 7*: unstimulated saliva samples from 37 active BD patients (*N* = 37) (age: 20–35 yrs, *μ*_age_ ± SD: 27.2 ± 6.3 yrs) with disease duration of 1–14 yrs (6.7 ± 3.2) were collected. The patients were males (males to females ratio = 1.92, *p* = 0.255, for total patients who visited the Rheumatology and Rehabilitation Unit (RRU) in FD-DU since 1990). Male genders and young age at BD onset currently remain the most reliable known predictors of the severe disease. Likewise, male gender is the main factor associated with mortality in BD and is reported to markedly influence BD expression and course, so that males usually tend to have more flare of BD compared to females. The patients of this level had confirmed at least one clinical sign according to the International Study Group for BD (ISG) criteria [21]. Inclusion criteria for patients of this level were lack of other oral or systemic disorders, not using orthodontic apparatuses, and no colchicine medications in last month. Other complementary experiments were adopted to check any changes of the biochemical and rheological limitations among inactive (*N* = 19) (age: 20–35 yrs, *μ*_age_ ± SD: 29.4 ± 4.7 yrs) and active (*N* = 37) (age: 20–35 yrs, *μ*_age_ ± SD: 27.2 ± 6.3 yrs) BD. The discrimination between active and inactive patients was confirmed by Behçet Syndrome Activity Score (BSAS). The BSAS scores were between 0 and 100 (0 represented inactive disease).

BD Current Activity Form (BDCAF) or named as Leeds Activity Inventory (range 0–12) was used considering the observed clinical signs and symptoms over a predefined time interval (28 days). Composite index (CI) and pain score (PS) were evaluated by visual analogue scale (VAS) in ranges of 0–10 and 0–100, respectively. Moreover, Rasch analysis was implemented in order to identify the clinical features. The Rasch analysis model assumes that the probability of a particular patient affirming a given item or category is a logistic function of the severity of the item and the activity of the patient's disease [22]. Due to the number of significance tests undertaken within each analysis, a significance level of *p* < 0.05 has been implemented. More information about the methods used for blood biochemical analyses are presented in the Supplementary Material (see Supplementary Material available online at https://doi.org/10.1155/2017/9596202).

### 2.5. Assessment of Oral Health

To determine oral health status, oral region of each participant was first examined, and the DMFT index was calculated and recorded for each of them. DMFT index measures the amount of permanent tooth decayed, missing, and filled in individual's mouth, ranging from 0 to 32.

### 2.6. DM Caries Study

A panoramic X-ray study was made. Observations corresponding to each individual were recorded on the odontogram, with the determination of CAO index for assessing carried, absent, and obturated teeth. Oral hygiene was rated based on the O'Leary plaque index (PI) [23]. Bacterial plaque was dyed and sampled with a blunt-tipped dental probe, sliding the latter along the gingival sulcus at four points per tooth and evaluating all surfaces. The presence or absence of plaque was estimated; regardless of its amount, the corresponding index was obtained as a percentage on summing the results and then dividing by the total number of points explored.

### 2.7. Sample Collection

Each individual gave at least 15 mL of saliva in consecution in 7-8 days. These samples were collected in Salivette tubes after overnight fasting. The candidate, upon waking each time, had provided a sample exactly between 9.30 am and 12.30 pm to avoid circadian effects. Individuals were instructed to refrain from eating, smoking, and drinking coffee and tea for 90 min prior to saliva collection. During saliva collection in morning, each subject sat in a relaxed position with head in a slightly inclined forward pose, allowing saliva to accumulate on the floor of mouth, considering that first few milliliters of saliva were discarded. Then, resting whole saliva was collected for 10 min and every 1 min the volunteer expectorated oral fluid available in mouth into 10 mL preweighed Eppendorf plastic tube. The hyposalivation cut-off values were determined. Samples were divided into six portions to determine pH, SFR, DMFT index, glucose content, Ca^2+^ concentration, and Mg^2+^ concentration.

### 2.8. Safety Considerations

Samples were handled as potentially infectious and the general guidelines for work with acids had been respected.

### 2.9. Salivary pH Method of Analysis

pH of saliva sample is dependent on the level of dissolved carbon dioxide (CO_2_); thus for a true pH value and to avoid any time-relating pH changes or loss of CO_2_, the degree of acidity was measured immediately after each sample collection, using a hand-held pH meter. Every day during the time of this research, pH meter had been calibrated with reference buffers of pH 4.0 and 7.0.

### 2.10. Glucose Assessment

Patients and control subjects were asked to give 5 mL venous blood, 2 mL of which was collected in ethylenediaminetetraacetic acid- (EDTA-) containing blood collection tubes and stored. The rest of blood for each sample was collected in a sterilized glass test tube.

Patients were also asked to wash their mouths with tap water and spit two or three times, after which they were informed to spit the saliva pooled in their mouths for the following 10 min into the sterile sample collection container.

Saliva and blood samples were centrifuged and glucose was estimated in the serum and supernatant saliva by the glucose oxidase (GOx) method using 4-aminophenazone (C_11_H_13_N_3_O) as oxygen acceptor [24]. The upper limit of fasting glucose concentration in serum was considered normal at 100 mg/dL (5.55 mM) [25]. The function of salivary glucose to serum glucose levels was identified.

### 2.11. Glycosylated Hemoglobin (HbA_1c_) Estimation

Blood samples of DM patients (Levels 4–6) and control subjects (Levels 1–3) were collected in EDTA tubes in order to subject them to HbA_1c_-level estimation, using ion exchange high-performance liquid chromatography (HPLC) method [26]. HbA_1c_ values were compared between DM patients and controls. Then, the function of salivary glucose to HbA_1c_ was identified.

### 2.12. Analysis Protocol of Earth-Alkaline Ions

**Figure 1:**
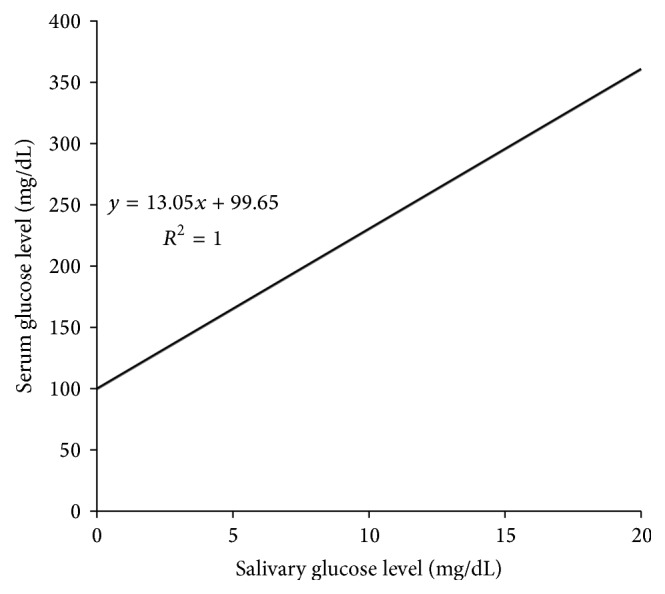
Correlation between preprandial salivary and serum glucose levels in DM study group (*p* < 0.001).

**Figure 2:**
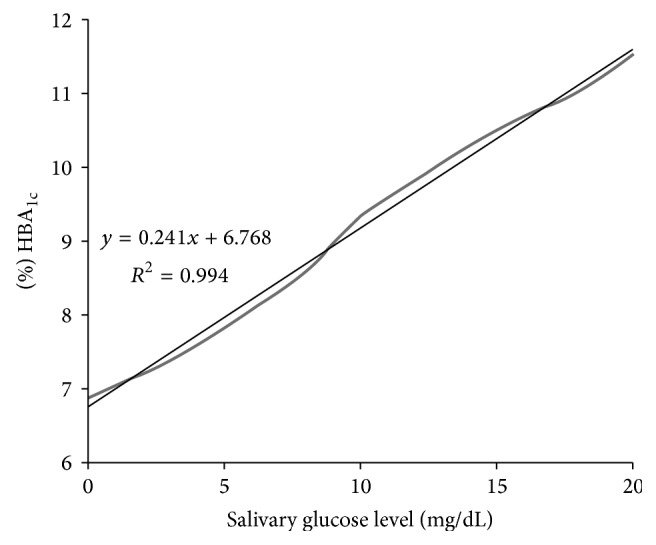
Correlation between preprandial salivary glucose levels and glycated hemoglobin (HbA_1c_) percentages in the study group (*p* < 0.001).

The analyses of Ca^2+^ and Mg^+2^ were based on ion-selective electrodes (ISE) technique. This technique has a high sensitivity which directly measures free ions in saliva. The methods for the separate determination of Ca^2+^ or Mg^2+^ using eight-point calibration curve and eight different standards for each ion with increasing concentrations (I–VIII) were used. Linear regression of the results for standards encompassing a series of samples was applied for the estimation of their contents of major earth-alkaline ions. After that, the concentrations of samples were derived by interpolation on the linear regression curve (Figures  1S and 2S) of the standards encompassing samples.

### 2.13. Sample Preparation for Earth-Alkaline Measurements

Samples were treated with 20% trichloroacetic acid (TCAA: Cl_3_CCOOH) (0.5 mL of 20% TCAA to 1 mL saliva sample) and centrifuged to remove proteins. The mixture (saliva-TCAA) was inserted the microcentrifuge and applied to a speed of 2500 rpm for 20 min to take out food rests, bacteria, mucosal cells, microorganisms, and desquamated cells from oral epithelium, protein, and other extraneous particles. Then, each filtrate was passed through a 0.45 *μ*m MF-Millipore to take away any existed higher-molecular-mass proteins and sulfates (SO_4_^2−^) in the matrix of sample. At this stage, pH was measured by pH meter (*n* = 3). The resulting solution was not diluted. 15 mL from each individual was guided prior to sample preparation to analyze the two earth-alkaline cations. Sodium hydroxide (NaOH, 1 mM) solution was added to adjust pH of specimen to about 11 where the mixture becomes clearer and more transparent. After that, saliva samples were divided into 10 portions (2 cations measurements × 5 repetitions) which fit the lab requirements for Ca^2+^ and Mg^2+^ analyses. Samples were stored in sealed Thermo Scientific Nalgene LDPE bottles away from direct sunlight at −20°C (−4°F) to avoid any significant change of enzyme activity (i.e., amylase). Immediately before analysis, samples were thawed at room temperature 25°C (77°F).

### 2.14. Statistical Analysis

Mann–Whitney *U* test was used to statistically compare the groups, with significant set at *p* < 0.05. Statistical analysis was performed with SPSS, version 16.0.2 (SPSS Benelux BV, Gorinchem, Netherlands), and Evalkit, version 3.1 (Tilburg, Netherlands), used for regression procedure according to Passing–Bablok analysis.

## 3. Results and Discussions

### 3.1. Salivary Glucose versus Blood Glucose

The salivary glucose levels showed a statistically strong significant association with very large correlation with blood glucose levels, suggesting that salivary glucose levels can be used as a monitoring tool for predicting glycemic control in diabetic patients (Figure 1). The growing in salivary glucose levels with increase in blood glucose levels has been suggested to be attributed to “leakage” across the basement membrane of the glands, particularly the parotid gland, when blood glucose levels rise beyond a threshold value.

### 3.2. HbA_1c_ versus Salivary Glucose

HbA_1c_ reflects the average blood glucose concentration over an extended period of time and remains unaffected by short-term fluctuations in blood sugar levels. A positive linear and very large correlation between preprandial salivary glucose concentrations and HbA_1c_ levels for the diabetic participants in this study was presented in Figure 2.

### 3.3. Biochemical Analysis

All reported data for control and illnesses of pH, SFR, DMFT, glucose, Ca^2+^, and Mg^2+^ were illustrated as means ± standard deviation ($\overset{-}{x}\pm \sigma$) in Table 1.

#### 3.3.1. Level 4 (TIDM)

TIDM patients showed many buccal disorders, increased gingival recession, and periodontal infections in addition to concomitant kidney diseases. Twenty-one of this group (22.1%) complained of dry mouth (xerostomia). Dehydration found was escorted by salivary glands structural changes. Hyposalivation (<0.2 mL/min) with no dehydration complaints was also diagnosed for this level (mean = 27, median = 27, 28.4%, *p* = 0.041). A similar finding was also reported by M. W. J. Dodds and A. P. Dodd [28] and Meurman et al. [29] suggesting the presence of diabetes-induced impairment of salivary gland function. By hand, it had been noticed that salivary glands were also affected directly or indirectly. Similar findings have also been previously described in the literature and were associated with diabetes-induced neuropathic changes in the salivary parenchyma with lymphocytic gland infiltrate as the one occurring in the pancreas of DM patients [30]. In other patients, dehydration and peripheral neuropathy were not observed in the current study.

Salivary fasting glucose concentration and HbA_1c_ were 10.28 ± 5.26 mg/dL (serum glucose: 216.2 ± 119.35 mg/dL) and 8.92 ± 2.72%, respectively. 38% of individuals of this level realized HbA_1c_ > 10% (*p* < 0.05) with higher limits followed by increasing of hyposalivation and glucose levels. Mean of glucose for TIDM patients was 89.2% significantly higher than controls (Level 1), indicating poor metabolic control of DM. These observations were similar to those reported by López et al. [31] and Siudikienè et al. [32].

Unstimulated SFR of TIDM patients was 67.1% lower than controls with no significant differences (Table 1) which meets the findings of Belazi et al. [33] and Miralles et al. [34]. The slop of TIDM patients' SFR (<0.5 mL/min) suggests salivary gland hypofunction [35] and this is an indication of high caries risk [36].

Salivary pH of patients of this level was significantly reduced 13.5% compared to controls, caused by lower SFRs which refers to a reduction of the buffer salivary capacity, microbial activity, and increasing of caries risk.

Compared to controls, Ca^2+^ decreased 1.56 times and showed no significant difference with controls but a positive correlation with glucose in saliva (*N* = 95, Pearson's *r* = 0.19). These findings meet with López et al. [31] and Mata et al. [37] ([Ca^2+^] > 1.25 mM). The decreasing of salivary Ca^2+^ concentrations of these patients was compensated by relatively Mg^2+^ increasing concentrations (1.10 times of corresponding value of controls). A positive correlation was detected between salivary Mg^2+^ and glucose (*N* = 95, Pearson's *r* = 0.16, *p* < 0.05). These results indicate that TIDM can lead to marked dysfunction of secretory capacity of salivary glands.

Although the literature [37] did not describe a consistent relationship between TIDM and dental caries, in the current study the high prevalence of caries with 68% carious lesion and significantly higher DMFT (5.5 times of controls' DMFT) are expectedly due to the increased concentration of salivary glucose (9.3 times of control's glucose) [38], higher acidity of the oral cavity, reduced SFR, and salivary gland dysfunction [39]. The prevalence of caries in TIDM patients was found in different locations particularly in the roots and dental neck regions.

DMFT, gingival index (GI), and PI were significantly (*p* < 0.001) 6.83% lower, 35.1% higher, and 15.4% higher than the findings of López et al. [31] which indicate the need of applying added measures with TIDM patients.

From Table 2 little differences of HbA_1c_ (22%), CAO (36.6%), GI (37.8), and PI (36.9) between TIDM patients and controls have been registered.

Consequently, patients of TIDM children are advised to control their sons' diets that can be a positive aspect. Dentists are recommended to study the counteracting factors of the minerals beneficial effects as dental plaque accumulation and immune factors contain in saliva when patients have poor salivary parameters. In addition, adequate oral hygiene program with exposure to fluoride (F^−^) (1,000–1,500 ppm) [40] and frequent use of artificial saliva can be of utmost importance for controlling the development of caries in TIDM patients.

#### 3.3.2. Level 5 (TIIDM, 20–39 yrs) and Level 6 (TIIDM, 40–60 yrs)

Hormonal, microvascular, and neuronal changes were noticed between TIIDM patients probably as a result of dysregulation that often compromises the ability of the multiple organ systems in functioning [44]. Prolonged hyperglycaemia in patients of these levels compromised the immune, cardiovascular, and renal and ophthalmic systems, which produced an array of complications like neuropathy, peripheral vascular, renal, retinopathy, and coronary heart diseases. The hyperglycemic environment reduces tissue growth by fibroblasts and osteoclasts. These tissues are weaker and delay wound healing [45]. The frequent observed of thirst and dry mouth could be results of poor glycaemic control in diabetics, which, in turn, associated with increased diuresis and fluid loss. These findings were also noticed by Cherry-Peppers et al. [46] who compared SFR of well-controlled TIIDM subjects and other individuals without diabetes. Oral health complications associated with TIIDM, were largely noticed by practitioners including xerostomia, tooth loss, gingivitis, periodontitis, odontogenic abscesses, and soft tissue lesions of the tongue and oral mucosa and these were agreed by Ben-Aryeh et al. [47]. Also, hypofunction was encountered in this type of diabetics due to the microvascular changes, hormonal imbalances, and autonomic neuropathies or a combination of these [48].

SFR (compared to controls of Level 2) was significantly lowered (31.8%) at Level 5 patients and insignificantly reduced (compared to controls of Level 3) (27.2%) at Level 6 patients, as a result of the microvascular complications and the autonomic neuropathy. This result relatively meets with Newrick et al. [49] (*N* = 8, 32–78 yrs, nondiabetic (ND) SFR = 0.68 mL/min, TIIDM SFR = 0.55 mL/min).

Salivary pH was insignificantly diminished (5.83%) for Level 5 patients and significantly lessened (18.0%) for Level 6 participants (Table 1). This refers to the acidogenic condition for carious lesions. Saliva exerts its major influence on caries initiation by means of plaque formation rather than by direct contact on the tooth surface; this causes plaque pH fall greater in dental-caries-susceptible subjects as also noticed by Chalmers [50] and Chu et al. [51]. Acidic pH was further presented in diabetic subjects by López et al. [31] correlated with the decrease of SFR and was attributed to either microbial activity or decrease in bicarbonate (HCO_3_^−^). Nevertheless, still fewer literature studies pertaining to salivary pH changes in TIIDM are available [52]. As a result, the acidic saliva and low SFR aggravated the tooth decay and caries inhibition processes [51, 53].

Biochemical determinations showed significant differences between diabetic Levels 5 and 6 and controls except for Mg^2+^. Study results showed significantly increased levels of salivary glucose (77.7% and 82.6% for Levels 5 and 6, resp.) in diabetics that meet with López et al. [31] who further found a negative correlation between salivary glucose levels and each of glycaemic status and HbA_1c_ levels of the subjects.

The increased levels of salivary glucose could be attributed to the altered glucose homeostasis where salivary glands act as filters of blood glucose (Level 5: 233.8 ± 128.7 mg/dL and Level 6: 274.1 ± 151.5 mg/dL) that would be changed by hormonal or neural regulation [54]. The high salivary glucose in conjunction with overall diminished SFR is responsible for the complaint of dry mouth and may contribute to the susceptibility to oral infections as periodontal disease and dental caries [55].

DMFT index (Table 1) was found significantly higher in diabetic patients as compared to controls. Furthermore, HbA_1c_, CAO, GI, and PI of TIIDM patients were significantly (*p* < 0.001) higher than controls (Table 2).

A positive correlation between Ca^2+^ and salivary glucose concentration is noticed. Ca^2+^ was significantly decreased (58.2% and 85.8% at Levels 5 and 6, resp.), probably since this earth-alkaline ion in oral environment is governed by the dissolution behavior of its salts and crystals found in forms of phosphates and hydroxides (OH^−^) in the enamel apatite lattice. The inadequate Ca^2+^ may alter the balance between the extracellular and intracellular *β*-cell calcium pools, which may interfere with normal insulin release, especially in response to a glucose load [56]. Thus, it is essential to maintain the homeostasis of Ca^2+^ that binds with phosphorous (P) which promotes bone mineralization. Bischoff-Ferrari et al. [57] and Holick [58] suggested vitamin D with calcium administration as modifiers of diabetic risk which maintain Ca^2+^ homeostasis and further proved nonskeletal outcomes including neuromuscular function and falls, psoriasis, multiple sclerosis, colorectal, and prostate cancer.

Mg^2+^ decreased insignificantly in diabetics (19.1% and 52.4% at Levels 5 and 6, resp.) possibly because of the important role of Mg^2+^ in glucose metabolism [59]. TIIDM patients have medical conditions reducing Mg^2+^ absorption from the gut and increasing losses from the body so they are at risk of magnesium inadequacy especially after 40 yrs old from Table 1. Therefore, it is particularly expected that TIIDM patients with insulin resistance increase urinary-Mg^2+^ excretion. The subsequent magnesium inadequacy might impair insulin secretion and action, thereby worsening diabetes control [60]. In light of the presented results, we recommend longitudinal clinical trials to examine the potential effects of liquid supplemental magnesium as magnesium chloride (MgCl_2_) solution on control of TIIDM.

The two bivalent ions (Ca^2+^ and Mg^2+^) exist in serum and are in continuous exchange phase with saliva over the dental plaque. This process is responsible for the “pool” or “reservoir” of calcium and magnesium in dental plaque and also maintains their saturations. Calcium observations (Level 6) were lower than Prathibha et al. [52] (*N* = 30, 40–55 yrs, nondiabetic [Ca^2+^] = 3.2 ± 0.25 mM, TIIDM [Ca^2+^] = 2.11 ± 0.06 mM); however we did not find a suitable study for Mg^2+^ comparison.

#### 3.3.3. Level 7 (Behçet Disease)

Clinical features of BD were significantly dominated by mucocutaneous involvement (oral and genital ulcers) and vascular association (venous to arterial: 3.2) which threatened patients' lives. In order to check BD activity, the histories of the clinical features have been studied. At that point, we defined the disease as active when symptoms and/or signs appeared in at least two to three organ systems. Exclusively, we registered 3-days acute uveitis (21.09%).

By and large, patients of this level were suffering from severity symptoms who presented oral ulcers, uveitis, blurry vision, punctate lesions through CNS, vitritis, incontinence, asymmetrical lower extremity hypertonia, papillitis, and spastic gait (Table 3).

The overall disease activity (BSAS: 32.5 ± 13.8; BDCAF: 5.0 ± 3.23; *r* = 0.436, *p* < 0.0001) was decreased (4.85%) with senior patients (>30 yrs) which reflected poorly immunosuppressive medications (13.8%). The CI and PS were very high for BD (Table 4) and the CI was correlated with BSAS score (*r* = 0.29, *p* = 0.03).

The observations of active BD data compared to inactive BD showed significant increasing of red blood cell distribution width (RDW) (Table 5) which is seemingly related to the decrease of homocysteine levels and hematimetric indices for active BD patients. These indices are mean corpuscular volume (MCV), mean corpuscular hemoglobin (MCH), and mean corpuscular hemoglobin concentration (MCHC). The increasing RDW is associated with the risk of thrombosis (43% of active BD patients with the majority of venous thromboses). Moreover, when patients had had previous uveitis, their rheological variables were compared with those for inactive disease. A significant growing of the rheological variables such as fibrinogen (Fbg), plasma viscosity (PV), erythrocyte aggregation at stasis (EA0), erythrocyte aggregation at 3 s^−1^ (EA1), and blood viscosity corrected hematocrit, 230 s^−1^ (BVc230 s^−1^), have been enrolled.

Thromboses were located in the lower limbs (21.7%), the iliac and caval vein plus pulmonary embolism (5.41%), the cerebral sinus (5.41%), the intracardiac area (2.70%), the upper limbs (2.70%), and the lungs with associated ischaemic stroke (2.70%). Eight patients had suffered repeated episodes of superficial phlebitis; six had more than one deep vein thrombotic episode and were therefore on long-term oral anticoagulant therapy with acenocoumarol (C_19_H_15_NO_6_, coumarin similar to warfarin) after monitoring hematocrit, hemoglobin, international normalized ratio, and liver panel. Despite prothrombotic mechanisms being not clear, a noticeable increasing erythrocyte aggregation has been registered among BD patients.

On the other hand, Table 4 presented the scores of periodontal indices of BD patients. We noticed clearly that about 10% of active BD patients were complained from poor oral health, poor prognosis for natural dentition, an increased number of extracted teeth due to multiple carious lesions, and changes in oral pH. In addition an increased incidence of tonsillitis and aggravation of disease by dental treatment were found in these patients. These findings showed that oral health was completely impaired in BD and associated with disease severity. Besides, these have clearly proved that saliva plays a critical role in the maintenance of oral health.

On the other hand, Table 1 had illustrated active BD's increasing ratios of pH and DMFT by 9.87% and 58.2%, respectively. The pH value for active BD in this study is 10% higher than the corresponding values obtained by Yosipovitch et al. [62]. However, inactive BD's pH (7.45 ± 0.90; *p* = 0.001 compared to controls) was less alkaline. DMFT values of this group in the current study are higher than Mumcu et al. [63] relative outcomes (DMFT: 7.2 ± 5.9). In comparison with active BD with controls, SFR, glucose, Ca^2+^, and Mg^2+^ decreased by 30.8%, 51.5%, 91.8%, and 95.8%, respectively. Compared to controls, inactive BD had lower limits of glucose (10.2%), Ca^2+^ (23.1%), and Mg^2+^ (35.2%). The daily frequencies of tooth brushing for active BD and inactive BD were 0.7 ± 0.4 and 0.9 ± 0.3 less than of controls (1.8 ± 0.8), albeit there were significant differences between patients' findings and controls (*p* < 0.05) with just one exception with SFR (*p* = 0.539).

The relatively decreased SFR is due to the increased DMFT that expected to play a vivid role in the pathogenesis of BD. This was clearly seen with altered taste sensation, oral candidiasis, and the growing number of caries. In addition, we believe that the decreasing SFR was directly related to the number of oral ulcers even though this observation was not registered or proved yet before. The lower SFR (12.9%) and the increasing of DMFT (36.5%) for the active disease compared to inactive disease have represented risk factors for the colonization of microorganisms (i.e.,* S. mutans*) in the oral environment (Table 1). This suggests the need for checking multiple etiological factors in disease pathogenesis and requires antibacterial treatment.

Unfortunately, we could not identify any studies concern with SFR, Ca^2+^, and Mg^2+^ in BD therefore; the current study recommends cell biology researchers doing more researches to investigate the main reasons that minerals in BD patients have such difficulties passing through cell membranes which make Ca^2+^ and Mg^2+^ not “bioavailable.”

As noticed from the biochemical analysis of BD patients, these are proved highly important in the maintenance of oral health.

However, to realize both satisfactory prevention and therapy of the impaired oral health of BD patients, according to the results of SFR and DMFT, we conclude that is necessary for those patients to brush their teeth twice a day for about 2 min and visit regularly dentists to check up their oral health. In addition, patients are advised to balance their comestibles rich in Ca^2+^ and Mg^2+^ to compensate their losses in the body. This procedure is highly important since the two minerals are antagonists, so having too much Ca^2+^ in the patient's diet will cause them to lose Mg^2+^ and vice versa. However, since systemic major organ involvement may result in life-threatening outcomes, an early diagnosis is recommended so that with appropriate immunosuppressive and biological treatment the chances of mortality and morbidity can be lessened.

## 4. Conclusion

Whole saliva is easily available oral fluid, reliable and a noninvasive diagnostic medium; thus, it was used in this study for the diagnosis of types I and II diabetes mellitus in well-controlled diabetic patients and* Behçet disease*. Furthermore, the salivary analysis in this work is proposed for the evaluation of systemic disorders which directly or indirectly affect salivary glands. The functions of these glands have influenced the quantity as well as the composition of saliva. Results showed that the adopted analyses are valuable for children and older adults, since collection of this fluid is associated with fewer compliance problems compared to blood compilation. Our data support the view that an appropriate evaluation of salivary clinical parameters, such as salivary flow rate, DMFT index, glucose, and electrolytes (i.e., Ca^2+^, Mg^2+^), is recommended when assisting diabetic and* Behçet disease* patients. At last, we expect that restoring the balance of BD oral microbial community mainly at the ulcer sites by probiotic therapy may heal oral ulceration. Further, we recommend doctors to order biochemical analysis of saliva concentrating on pH, salivary flow rate, DMFT, glucose, and earth-alkaline ions for early detection of systemic diseases, especially for metabolic and vasculitic patients.

## Supplementary Material

A wide array of the salivary biochemical (incl. Ca^2+^ and Mg^2+^) analysis and the pertinent analytical parameters as the ions detection limits and the recoveries of the optimized methods measurements by ion selective electrode (ISE), in addition to the blood and plasma microbiological analyses for Behçet's Disease are presented in this paper which suggests saliva as a proper biological indicator.

## Figures and Tables

**Table 1:** pH, SFR, DMFT, glucose, and electrolytes of whole saliva from patients with TIDM, TIIDM, and *Behçet* disease compared with control groups.

Level	pH	SFR (mL/min)	DMFT index	Glucose (mg/dL)	Ca^2+^ (mM)	Mg^2+^ (mM)
1	7.10 ± 0.72	0.82 ± 0.24	0.99 ± 0.72	0.96 ± 0.31	1.84 ± 0.64	0.15 ± 0.02
2	7.03 ± 0.70	1.07 ± 0.39	4.25 ± 0.19	2.29 ± 0.72	2.20 ± 0.07	0.47 ± 0.05
3	7.22 ± 0.75	0.92 ± 0.31	4.68 ± 0.28	2.33 ± 1.31	1.97 ± 0.43	0.21 ± 0.03
4	6.14 ± 0.42 (0.003)	0.27 ± 0.12 (0.056)	5.46 ± 1.82 (0.005)	8.93 ± 4.47 (0.011)	1.48 ± 0.39 (0.069)	0.23 ± 0.03 (0.055)
5	6.62 ± 0.46 (0.053)	0.73 ± 0.26 (0.037)	8.38 ± 0.28 (0.012)	10.28 ± 5.26 (0.005)	1.16 ± 0.39 (0.026)	0.38 ± 0.07 (0.064)
6	5.92 ± 0.32 (0.000)	0.67 ± 0.21 (0.065)	14.18 ± 3.25 (0.017)	13.37 ± 6.97 (0.003)	0.28 ± 0.01 (0.014)	0.10 ± 0.02 (0.057)
7^(1)^	7.80 ± 0.96 (0.001)	0.74 ± 0.29 (0.539)	10.16 ± 5.44 (0.028)	1.11 ± 0.42 (0.013)	0.18 ± 0.05 (0.033)	0.02 ± 0.00 (0.024)

Differences of distributions in the two groups (patient-control) are presented as critical values for Mann–Whitney *U* test in parentheses; level of *significance*: 5% (*p* = 0.05).

^*(1)*^ *Streptococcus mutans (S. mutans)* (74.6% in patients with active oral ulcers versus 25.4% in inactive ulcers; *p* = 0.008) in the whole saliva was remarkably associated with disease severity, oral ulcers, and DMFT. *S. mutans* is a major etiology factor for the development of dental caries and is a member of oral biofilm [27]. Furthermore, no significant difference was observed in the healing time of oral ulcers for active (7.8 ± 1.4 days) and inactive (7.5 ± 2.2 days) diseases.

**Table 2:** Comparison of clinical variables in diabetic group versus controls.

Medical state (level)	CAO^(2)^	GI^(3)^	PI^(4)^	HbA_1c_^(5)^
Control 1 (Level 1)	5.17 ± 3.72	0.46 ± 0.12	0.41 ± 0.18	6.96 ± 2.03
TIDM (Level 4)	8.15 ± 4.59	0.74 ± 0.16	0.65 ± 0.26	8.92 ± 2.72
*Student's t-test*	2.483	0.592	0.611	0.332
Control 2 (Level 2)	6.90 ± 4.73	0.62 ± 0.14	0.57 ± 0.22	7.20 ± 2.12
TIIDM (Level 5)	14.6 ± 8.16	1.38 ± 0.27	1.19 ± 0.46	9.25 ± 3.41
*Student's t-test*	2.749	0.706	0.735	0.402
Control 3 (Level 3)	7.18 ± 4.01	0.67 ± 0.18	0.63 ± 0.30	7.33 ± 2.15
TIIDM (Level 6)	15.4 ± 8.37	1.76 ± 0.44	1.58 ± 0.83	10.0 ± 3.74
*Student's t-test*	3.119	0.782	0.806	0.445

$\overset{-}{x}\pm \sigma$: mean ± standard deviation.

Statistical significance (*p* < 0.05).

^(2)^Method developed by Knutson [41].

^(3)^Method developed by Löe [42].

^(4)^Method developed by Silness & Löe [43].

^(5)^Method developed by Flückiger & Mortensen [26].

**Table 3:** Observatory clinical signs and symptoms associated with Behçet's disease.

	*χ* ^2^		Frequency (%)
	Residual	Probability	
Disease activity (patient)	−2.11	0.05	
*Oral ulcers*	*0.92*	*0.63*	*86.2*
Uveitis	0.70	0.51	72.8
*Hypertonia*	*0.39*	*0.24*	*33.8*
Spastic gait	0.27	0.22	28.4
*Blurry vision*	−*0.11*	*0.18*	*25.5*
Incontinence	−0.17	0.12	16.7
*Punctate lesions through CNS*	−*0.23*	*0.11*	*13.8*
Papillitis	−0.26	0.10	11.6
*Asymmetrical lower extremity hypertonia*	−*0.34*	*0.09*	*9.2*
Vitritis	−0.39	0.08	4.9
*Person separation index*	*0.64*		
Item-trait interaction	0.22		

*χ* ^2^: Chi-squared statistic.

Residual: standardized difference between the observed score and the expected score according to the model.

Person separation index: the estimate of the replicability of person placement that can be expected if the samples of persons are given another set of items measuring the same construct. Analogous to Cronbach's *α*, it is bounded by 0-1 range (i.e., >0.8 is very good).

Item-trait interaction identifies the degree of the overall fit of the measure to the model. It assesses the degree to which the measure is diverging from the model in a systematic way that is not accounted for by chance alone.

**Table 4:** Periodontal test outcomes of *Behçet* disease patients.

Investigation	Findings
Disease duration (yr)	8.2 ± 5.9
GI^(6)^	2.9 ± 0.7
Number of carious teeth	2.7 ± 2.6
Number of extracted teeth	5.3 ± 5.5
Oral ulcers (number/month)^(7)^	4.6 ± 6.2
PI^(8)^	2.8 ± 0.8
Sulcus bleeding index (SBI)^(9)^	3.1 ± 1.0

^(6)^Method developed by Löe [42].

^(7)^CI = 8.3 ± 1.7; PS = 60.7 ± 24.5.

^(8)^Method developed by Silness & Löe [43].

^(9)^Method developed by Mühlemann & Son [61].

**Table 5:** Comparison of body mass index and biochemical and rheological parameters for inactive and active *Behçet* disease patients (*n* = 3).

Variable	Inactive patients (*N* = 19)	Active patients (*N* = 37)	*p*
Body mass index (BMI) (kg/m^2^)	22.8 ± 3.13	25.4 ± 3.52	0.609
*Elongation index at 60 Pa (EI60) (%)*	*57.22 ± 4.34*	*59.34 ± 3.17*	*0.602*
Plasma viscosity (PV) (cP)	1.18 ± 0.05	1.29 ± 0.10	0.001
*Blood viscosity native hematocrit, 230 s* ^−*1*^ * (BVn230 s* ^−*1*^ *) (cP)*	*4.02 ± 0.49*	*4.33 ± 0.47*	*0.401*
		*4.55 ± 0.49*	
Blood viscosity corrected hematocrit, 230 s^−1^ (BVc230 s^−1^) (cP)	4.35 ± 0.29	5.04 ± 0.43	0.008
*Red blood cell distribution width (RDW)*	*12.06 ± 0.58*	*16.14 ± 1.46*	*<0.001*
Mean corpuscular volume (MCV) (femtoliters/cell (fL))	90.38 ± 4.74	88.27 ± 5.16	0.311
*Mean corpuscular hemoglobin (MCH) (pictograms/cell (pg))*	*30.48 ± 1.42*	*29.53 ± 1.89*	*0.065*
Mean corpuscular hemoglobin concentration (MCHC) (%)	34.89 ± 0.91	34.22 ± 1.20	0.157
*Hematocrit (Htc) (%)*	*42.8 ± 3.58*	*39.2 ± 3.36*	*0.407*
Leucocytes (Leu × 10^9^/L)	6.53 ± 1.08	7.80 ± 2.95	0.095
*Erythrocyte aggregation at stasis (EA0)*	*2.75 ± 0.94*	*4.15 ± 0.95*	*0.001*
Erythrocyte aggregation at 3 s^−1^ (EA1)	6.63 ± 0.94	7.92 ± 1.20	0.003
*Fibrinogen (Fbg) (mg/dL)*	*250 ± 51*	*315 ± 97*	*0.001*
Total cholesterol (T-Chol) (mg/dL)	211 ± 37	209 ± 39	0.611
*Triglycerides (TG) (mg/dL)*	*90 ± 40*	*108 ± 49*	*0.243*
