# Supplementary material for: Salivary Distinctiveness and Modifications in Males with Diabetes and Behçet's Disease
Source: Biochem Res Int. 2017 Feb 21;2017:9596202. doi: 10.1155/2017/9596202 (PMC5339495; doi:10.1155/2017/9596202)
Supplement: Supplementary file 1 — A wide array of the salivary biochemical (incl. Ca2+ and Mg2+) analysis and the pertinent analytical parameters as the ions detection limits and the recoveries of the optimized methods measurements by ion selective electrode (ISE), in addition to the blood and plasma microbiological analyses for Behçet's Disease are presented in this paper which suggests saliva as a proper biological indicator. [file 9596202.f1.pdf]

Supporting information

Salivary Distinctiveness and Modifications in males with Diabetes and Behçet's Disease

Loai Aljerf,[1] Iyad Alhaffar,[2]

[1]*Department of Life Sciences, Faculty of Dentistry, University of Damascus, Damascus, Syria;*

[2]*Department of Oral Medicine, Faculty of Dentistry, University of Damascus, Damascus, Syria*

Correspondence should be addressed to Loai Aljerf; envirochrom@hotmail.com

1. Chemical s and Reagents

The following items show the commercial sources and some characterizations of purchased chemicals used in experimental section:

1.1. Calcium carbonate ($CaCO_3$), ReagentPlus, 99% (Sigma-Aldrich Chemie Gmbh, Munich, Germany)

1.2. Calcium standard solution (1000 mgCa^{2+}/L = 25 mM Ca^{2+}), Certipur, NIST $Ca(NO_3)_2$ in 500 mM HNO_3 (Sigma-Aldrich Chemie Gmbh, Munich, Germany)

1.3. Doubly distilled deionized water (dd-DI H_2O) (Milli-Q Ultrapure water specifications: 18.2 MΩ/cm @ 25 °C (77 °F), TOC ≤ 5 ppb, Bacteria ≤ 1 CFU/mL), which is non-absorbent under UV radiation, has been used throughout

1.4. Hydrochloric acid (HCl), BDH ARISTAR Ultra, 32-35% (VWR, Radnor, PA, USA)

1.5. Methyl red, acid-base indicator grade (Sigma-Aldrich Chemie Gmbh, Munich, Germany)

1.6. Nitric acid (HNO_3) 65%, ISO grade, ≥ 99.5% (Merck-Millipore Co., Massachusetts, USA)

1.7. Oxide-free magnesium ribbon, High quality grade, 99.9% (Sherman Chemicals Ltd, Gillingham, UK)

1.8. pH 4.0 and 7.0 buffer solutions (Sigma-Aldrich Chemie Gmbh, Munich, Germany)

1.9. Potassium chloride (KCl), ACS reagent, 99% (VWR, Radnor, PA, USA)

1.10. Potassium permanganate ($KMnO_4$), ACS reagent, 99% (Sigma-Aldrich Chemie Gmbh, Munich, Germany)

1.11. Sodium hydroxide (NaOH) anhydrous, ARG, ≥ 99% (Sigma-Aldrich Chemie Gmbh, Munich, Germany)

1.12. Standard Reference Material (SRM-2670), simulated freeze dried urine control sample (National Institute of Standards and Technology (NIST), Gaithersburg, USA)

2. Materials

Materials used in this study were listed as follows:

2.1. Desiccator nalgene, Thermo Scientific (Nalge Nunc Int., Rochester, NY, USA)

2.2. MF-Millipore membrane filter (0.45 μm, 47 mm) (Merck-Millipore Co., Massachusetts, USA)

2.3. Micromedic Model 2500 Automatic Pipette and Ependorf pipettes with disposable tips (Micromedics Systems, Division of Rohm and Haas, Horsham, PA, USA)

2.4. Reusable plastic beakers (Consolidated Plastics Inc., Stow, Ohio, USA)

2.5. Salivette tubes (Sarstedt AG & Co., Nümbrecht, Oberbergischer Kreis, Germany)

2.6. Thermo Scientific Nalgene low-density polyethylene (LDPE) bottles, (Cole-Parmer International, Vernon Hills, Illinois, USA)

2.7. Transparent microcentrifuge Eppendorf plastic tubes with attached cap lids (Jack Chen Biologix Plastics Co., Ltd., Shanghai, China)

3. Apparatuses

3.1. Force 7 micro- centrifuge (Denver Instruments, Norfolk, UK)

3.2. Microlyte 6 analyzer (Thermo Fisher Scientific Oy, Vantaa, Finland)

3.3. Panasonic Undercounter Laboratory Freezer (Panasonic Healthcare Corporation, Wood Dale, Illinois, USA)

3.4. Sartorius Cubis weighing balance (Sartorius AG, Gottingen, Germany)

3.5. Sentron pH system 1001 (Sentron Europe, Roden, The Netherlands)

3.6. Universal Oven UN30 (Memmert GmbH+Co. KG., Schwabach, Germany)

4. Reagents Preparation

4.1. Ca^{2+} stock solution (Std $_{stock,Ca^{2+}}$, 8 mg $CaCO_3$/mL = 80 mM $CaCO_3$ = 3.2 mg Ca^{2+}/mL = 80 mM Ca^{2+}): A primary standard grade of anhydrous $CaCO_3$ (FW=100.087) was dried in the oven at 105 °C (221 °F) for 90 min. 4 g (to 1 mg) of $CaCO_3$ were accurately weighed and transferred to 500 mL volumetric flask. 10 mL of dd-DI H_2O were slowly added with 1:1 HCl solution through a funnel to the volumetric flask until effervescence

ceased. That made all the CO_3^{2-} dissolved and the resulting solution was completely cleared out. At this stage, the addition of HCl had converted the $CaCO_3$ to $CaCl_2$. 250 mL dd-DI H_2O was added to the flask then this quantity had boiled for few minutes to expel CO_2. The solution was cooled and few drops of methyl red indicator were added. The solution was adjusted to the intermediate orange color by adding few drops of 1:1 HCl. At last, the solution was quantitatively transferred and diluted to the mark of the volumetric flask with dd-DI H_2O

4.2. Mg^{2+} stock solution (Std $_{stock,Mg}{}^{2+}$, 1.9 mg Mg^{2+}/mL = 80 mM Mg^{2+}): A primary standard solution was prepared by dissolving 1.9 g oxide-free magnesium ribbon (to 1 mg) in 5 mL HCl (S.G.1.18, 6 M) then diluted to the mark of the 1000 mL volumetric flask with dd-DI H_2O and mixed thoroughly

4.3. Ca^{2+} ISA (ionic strength adjustor), 1 M KCl: dd-DI H_2O was filled a 100 mL volumetric flask up to the halfway mark. 7.46 g (to 1 mg) of previously dried anhydrous KCl were added. The flask swirled gently to dissolve the solid, then, the flask filled to the mark with dd-DI H_2O, capped and upended several times to mix the solution

4.4. Mg^{2+} ISA, 4 M KCl: dd-DI H_2O was filled a 100 mL volumetric flask up to the halfway mark and 29.8 g (to 1 mg) of previously dried anhydrous KCl was added. The flask swirled gently to dissolve the solid then filled to the mark with dd-DI H_2O, capped and upended several times to mix the solution

4.5. $KMnO_4$ (25 mM): 3.95 g (to 1 mg) of $KMnO_4$ was added to 250 mL of dd-DI H_2O in a 1000 mL volumetric flask. The solution was stirred to dissolve then diluted to the mark with dd-DI H_2O

4.6. NaOH, (1 mM): 1 mL of NaOH (1 M) was diluted to 1000 mL with dd-DI H_2O

4.7. Glassware rinsing solution: All glass components were rinsed by soaking each of them in acidified (nitric-permanganate) solution so these components were treated at first by 50% HNO_3, washed by dd-DI H_2O, followed by (25 mM) $KMnO_4$ treatment; at last the glassware were rinsed several times again with dd-DI H_2O

5. Ca^{2+} and Mg^{2+} Measurements by ISE

ISE technique was implemented for Ca^{2+} and Mg^{2+} measurements using a new specific electrode equipped with a selective membrane of high surface tension (solid-state PVC polymer) for each cation. The membrane containing a known concentration of the earth-alkaline cation was in contact with an internal filling solution. As previously mentioned, Ca^{2+} selective electrode was based on solid-state PVC polymer using phenylene bis (ditolyl) phosphine oxide as ionphore, while, Mg^{2+} selective electrode was based on tetraphenylborate salt of Mg-4,7-diphenyl-1,10-

phenanthroline (1:3) complex in *o*-nitrophenyloctyl ether in PVC matrix. During analysis, the major earth-alkaline ions were associated with the membrane on each side so that the membrane develops an electric potential or electromotive force (EMF). The amount of the EMF was determined by taking the difference between concentrations of test solution and internal filling solution. The system had been calibrated every 24 hrs during time of measurements. The calibration procedure was required the use of standard solutions (low, medium, and high earth-alkaline concentrations) to determine slope factor.

Preliminary analysis of 20 saliva samples showed time response of calcium electrode 45 s and 65 s for magnesium electrode. Response time is the time taken for the potential to reach a value of 1 mV from the final equilibrium potential after an instantaneous change in the analyte concentration. Reproducibility of these measurements was found at 0.2 mV level and baseline stability obtained at 1 mV/h. However for practical purposes, 100 s waiting time was adopted for stable readings with both electrodes as a criterion in the analysis of saliva. 10 µL ISA was drawn anaerobically through a silicone-free tube to 500 µL of aliquot (blank, standard, or sample) and placed in a mini-polyethylene cup to raise pH of solution to an optimal pH environment for the specific ion being measured, eliminate the possibility of proton (H^+) interference, and avoid the diffusion potentials which could create transport barriers. pH solution was checked for each sample by the specific electrode of the analyzer and adjusted at 11 using potassium hydroxide (KOH, 1.25 mM) solution that gave an increasing of the earth-alkaline ions electrodes responses at preconditioning analytical stage. Then, 150 µL of the aliquot was inserted the apparatus within 3-5 min stabilization time and its potential was recorded. Data of measurements were analyzed using calculated mean of 5 replicates (n = 5). After that, ionic calibration series was prepared in terms of the potential of standard solutions as a function to logarithmic concentration (mg/L) of element. After defining the two calibration curves for Ca^{2+} and Mg^{2+} by ISE, standard addition technique was implemented for the measurement of these ions in oral fluid samples. Blank samples then biological specimens had been analyzed under the same conditions to maintain a constant ionic strength of the flow-through cell.

6. Calibration Series

A series of standards was prepared for each earth-alkaline element measurement by accurately pipetting the calculated volume of the corresponding standard stock solution ($Std_{stock,Ca^{2+}}$ or $Std_{stock,Mg^{2+}}$) then diluting each

aliquot to 2000 mL with dd-DI H_2O in suitable volumetric flask (Tables 1S and 2S). Eight standard solutions were employed to create a calibration curve for each of the two ions and three of them were marked as low, medium, and high quality controls (QCs: artificial saliva (Table 3S) and SRM-2670).

TABLE 1S: Ca^{2+} calibration series.

St_i Ca^{2+} (1)	St_1	St_2	St_3	St_4	St_5	St_6	St_7	St_8
$V_{0,i}$ (mL) (2)	5	12.5	27.5	37.5	52.5	65	77.5	97.5
$V_{final,Sti}$ (mL) (3)	2000	2000	2000	2000	2000	2000	2000	2000
St_i conc. (mM)	0.2	0.5	1.1	1.5	2.1	2.6	3.1	3.9
St_i conc. (mg/L)	8	20	44	60	84	104	124	156

(1) Eight standards of Ca^{2+} marked as: St_1, St_2, St_3, St_4, St_5, St_6, St_7, and St_8

(2) $V_{0,i}$ (mL): Volume of Std $_{stock,Ca}{}^{2+}$ (80 mM Ca^{2+} = 3.2 mg Ca^{2+}/mL)

(3) $V_{final,Sti}$ (mL): Final volume of Ca^{2+} standard solution (Std_i Ca^{2+}) after dilution

TABLE 2S: Mg^{2+} calibration series.

St_j Mg^{2+} (4)	St_9	St_{10}	St_{11}	St_{12}	St_{13}	St_{14}	St_{15}	St_{16}
$V_{0,j}$ (mL) (5)	2.5	5.0	7.5	12.5	15.0	17.5	22.5	25.0
$V_{final,Stj}$ (mL) (6)	2000	2000	2000	2000	2000	2000	2000	2000
St_j conc. (mM)	0.1	0.2	0.3	0.5	0.6	0.7	0.9	1.0
St_j conc. (mg/L)	2.4	4.9	7.3	12.1	14.6	17.0	21.9	24.3

(4) Eight standards of Mg^{2+} marked as: St_9, St_{10}, St_{11}, St_{12}, St_{13}, St_{14}, St_{15}, and St_{16}

(5) $V_{0,j}$ (mL): Volume of Std $_{stock,Mg}{}^{2+}$ (80 mM Mg^{2+} = 1.9 mg Mg^{2+}/mL)

(6) $V_{final,Stj}$ (mL): Final volume of Mg^{2+} standard solution (St_j Mg^{2+}) after dilution

TABLE 3S: Composition of the artificial saliva medium.

Reagent	Added mass (mg) to 1000 mL dd-DI H_2O	Cation concentration (mM)
$CaCl_2 \cdot 2H_2O$	227.8	1.55
$MgCl_2 \cdot 6H_2O$	57.56	0.25
KCl	963.9	12.9
KH_2PO_4	654.5	4.8
KSCN	971.81	10
NaCl	125.6	2.15
$NaHCO_3$	630.8	7.51
$NaNO_2$	690	10
$Na_2SO_4 \cdot 10H_2O$	763.2	2.37
NH_4Cl	178.0	3.33
Urea	200.0	3.3

The limit of detection (C_L) for the earth-alkaline ions measured by ISE was provided in Table 4S.

TABLE 4S: Limit of detection (C_L) values for Ca^{2+} and Mg^{2+} measurements.

Analyte (optimized method)	S_B (mg/L)	C_L (mg/L)
Ca^{2+} (ISE)	1.17	4.1
Mg^{2+} (ISE)	0.70	1.4

The recovery (R, %), relative standard deviation of percent recovery (S (R)), relative percent difference (RPD), instrument detection limit (IDL), method detection limit (MDL), and limit of quantitation (LOQ) have been implemented as important pieces of data which may demonstrate the importance of biases at different concentration levels.

TABLE 5S: Ca^{2+} and Mg^{2+} target concentration values (mM) in QC samples.

Analyte (optimized method)	True value, (mM)	Observed value, (mM)		R (%)	S (R)	RPD	IDL	MDL	LOQ
		$\overline{x}$	σ						
Ca^{2+} (ISE) [(7)]	1.55	1.48	0.0153	95.5	3.4	12.1	0.047	0.048	0.153
Ca^{2+} (ISE) [(8)]	0.75	0.71	0.0078	94.7	3.6	12.0	0.234	0.239	0.078
Mg^{2+} (ISE) [(7)]	0.25	0.24	0.0051	96.0	3.4	8.4	0.015	0.016	0.051
Mg^{2+} (ISE) [(8)]	0.87	0.83	0.0184	95.4	3.5	8.2	0.055	0.058	0.184

[(7)] Analyte in artificial saliva; standard error (SE, mg/L) for Ca^{2+} and Mg^{2+} are 0.072 and 0.019, respectively; bias (%) of Ca^{2+} and Mg^{2+} measurements are 4.32 and -1.98, respectively; maximum confidence intervals for Ca^{2+} and Mg^{2+} measurements are 1.23 and 1.25, respectively

[(8)] Analyte in SRM-2670; (SE, mg/L) for Ca^{2+} and Mg^{2+} are 0.055 and 0.0148, respectively; bias (%) of Ca^{2+} and Mg^{2+} measurements are 3.27 and -1.41, respectively; maximum confidence intervals for Ca^{2+} and Mg^{2+} measurements are 1.28 and 1.31, respectively

7. Optimization of Sample Preparation Method

Sample preparation was accommodated to get rid of the interfering factors (i.e. PO_4^{3-}, turbidity) that could weaken the response of the signal of the technique used for ion determination. Sample solutions were stored at -20 °C (-4 °F) in 5 mL polypropylene cryovials. After thawing, the solutions were only used during one measurement campaign

and then discarded after 2 freezing/thawing cycles. Besides, to define the best method selectivity, the comparison of the LOQ and linearity between the optimized methods has been adopted (Table 5S).

8. Calibration Curves

The calibration curves of Ca^{2+} and Mg^{2+} were presented in Figures 1S and 2S.

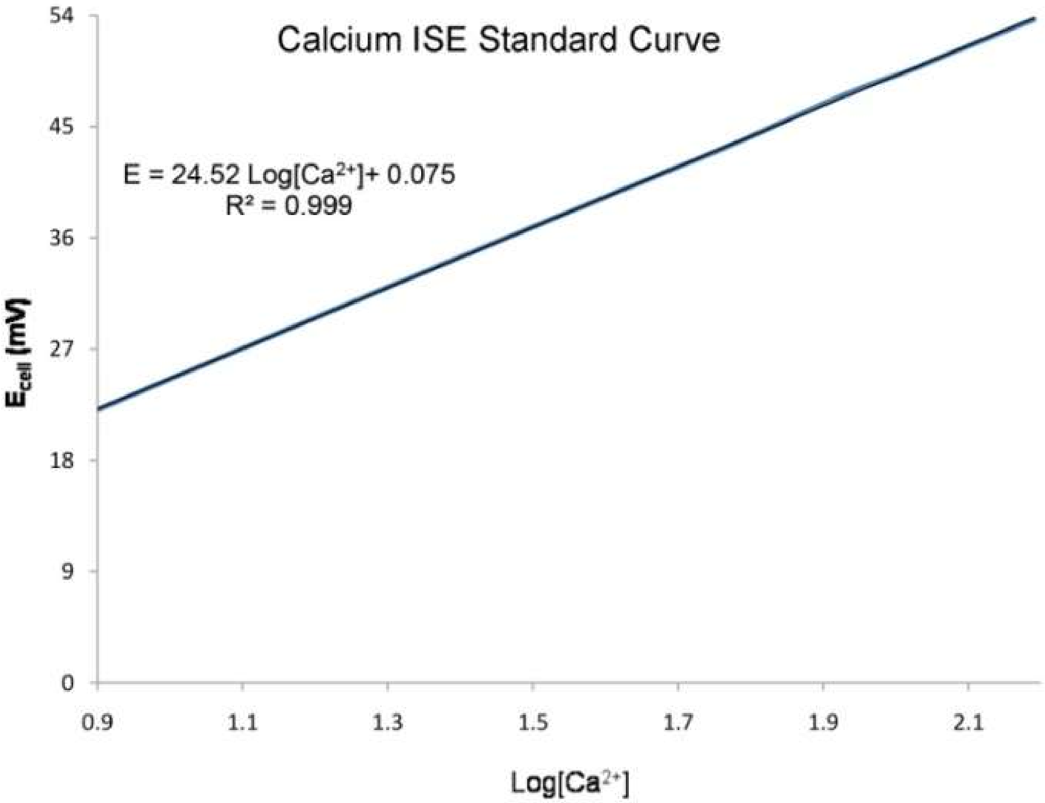

FIGURE 1S: Calcium calibration curve by ISE method: EMF vs. Log[Ca^{2+}].

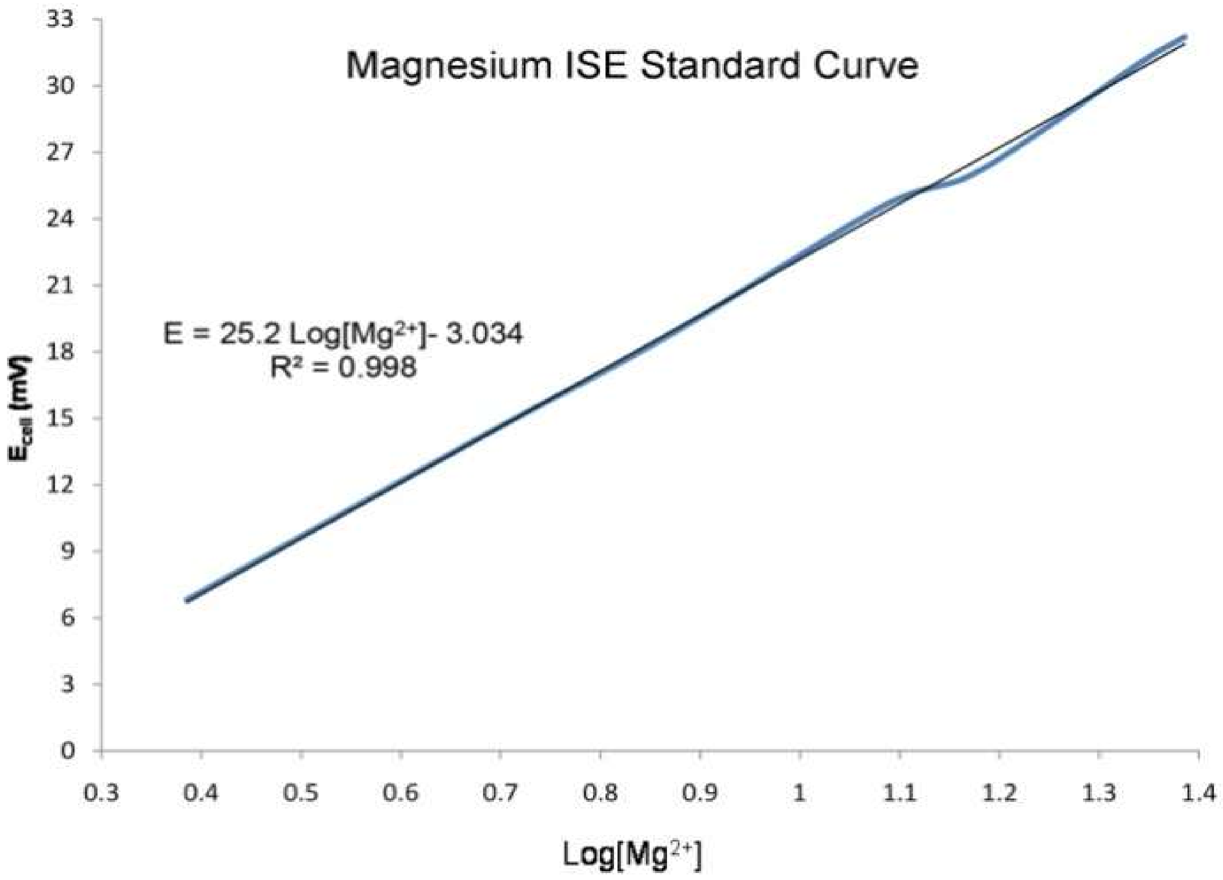

FIGURE 2S: Magnesium calibration curve by ISE method: EMF vs. Log[Mg^{2+}].

9. Microbiological Analysis

A CRT bacteria kit (Ivoclar Vivadent, Schaan, Liechtenstein) was used to determine *S. mutans* levels. Saliva samples were dropped on the CRT bacteria kit media by pipette. After incubation at 37 °C for 48 hours, viable colonies were measured. The salivary counts of *S. mutans* determined by the number of colony forming units (CFU) were measured semi-quantitatively.

10. Blood Biochemical Analysis

10.1. Methodology. Total cholesterol (T-Chol) and triglycerides (TG) were measured by enzymatic techniques in a Dax- 72 autoanalyzer (Bayer Diagnostic, Tarrytown, NY, USA). Fibrinogen (Fbg) was determined using coagulometric techniques in an ACL-7000 autoanalyzer (Instrumentation Laboratory, Milan, Italy). Hematocrit was assessed using a microcentrifuge at 15000 g for 7 min. Erythrocyte aggregation was measured in an erythrocyte aggregometer (Myrenne, Germany) at stasis (EA0) and at low shear rate of 3 s1 (EA1) after adjusting the hematocrit to 45% with autologous plasma [1]. Plasma viscosity (PV) was determined in a capillary plasma viscosimeter (Fresenius, Germany) at 37 °C [2]. Blood Viscosity (BV) was measured in a Brookfield model DV III viscosimeter (Brookfield Engineering Laboratories, Stoughton, MA, USA) for 230 s1 both at a native and a corrected hematocrit of 45%, at 37 °C. Erythrocyte deformability (EI) was determined in a shear stress diffractometer (Rheodyn SSD, Myrenne) at 12, 30 and 60 Pa [3]. Because of the high correlation coefficient between these three values [4], only the results obtained at 60 Pa are given. Basic blood cell count and erythrocyte indexes (MCV, MHC, MCHC) were determined using a Sysmex NE 8.000 autoanalyzer (TOA, Medical Electronics, Kobe, Japan).

10.2. Statistical Analysis. All continuous variables were evaluated for normality of distribution. TG values were markedly skewed and these data were logarithmically transformed before statistical analysis. χ^2 tests were used to compare differences in percentages between active and inactive patients. The data are expressed as means ± standard deviation (SD). A bilateral p value < 0.05 was considered statistically significant. All analyses were calculated using the Statistical Package for SPSS, version 16.0.2 (SPSS Benelux BV, Gorinchem, The Netherlands).

References

[1] H. Schmid- Schönbein, E. Volger, P. Teitel, H. Kiesewetter, V. Daver, and L. Heilmann, "New hemorheological techniques for the routine laboratory," *Clinical Hemorheology and Microcirculation*, vol. 2, no. 1-2, pp. 93-105, 1983. http://dx.doi.org/10.3233/ch-1982-21-212

[2] F. Jung, H. G. Roggenkamp, R. Scheider, and H. Kiesewetter, "Das Kapillarschlauch-Plasmaviskosimeter ein neues meßgerät zur quantifizierung der Blutplasmaviskosität (The Capillary Tube-Plasma Viscometer A New Instrument for the Measurement of the Plasma Viscosity)," *Biomedizinische Technik/Biomedical Engineering*, vol. 28, no. 11, pp. 249-252, 1983. http://dx.doi.org/10.1515/bmte.1983.28.11.249

[3] P. Ruef, J. M. B. Pfschl, and O. Linderkamp, "The rheodyn SSD for measuring erythrocyte deformability," *Biorheology*, vol. 32, no. 2-3, pp. 357-358, 1995. http://dx.doi.org/10.1016/0006-355x(95)92349-f

[4] H. Schmid- Schönbein, P. Ruef, and O. Linderkamp, "The shear stress diffractometer rheodyn SSD for determination of erythrocyte deformability I. Principles of operation and reproducibility," *Clinical Hemorheology and Microcirculation*, vol. 13, no. 2-3, pp. 447-457, 1993.
